# Effects of experimentally induced self-affirmation on the openness to meat reduction and alternative protein sources

**DOI:** 10.3389/fpsyg.2023.1021891

**Published:** 2023-04-21

**Authors:** Marija Branković, Anastasija Budžak, Nađa Tulić, Jovana Janković

**Affiliations:** ^1^Insitute for Philosophy and Social Theory, University of Belgrade, Belgrade, Serbia; ^2^Faculty of Media and Communications, Singidunum University, Belgrade, Serbia

**Keywords:** self-affirmation, meat consumption, alternative protein, group affirmation, self-threat

## Abstract

**Introduction:**

Consumption of animals entails disregarding the pain of sentient beings, and acknowledging this can threaten an individual’s image of oneself as a moral person. Also, abstaining from meat in a meat-eating culture can threaten an individual’s valued group identity. Previous research on inter-group relations suggests that self-affirmation, affirmation of personally or collectively important values, can help individuals alleviate self-threats since it enhances one’s global self-image and decreases threat perceptions.

**Methods:**

We tested for potential effects of self-affirmation on openness toward reducing meat consumption in an experimental study. Participants (*N* = 277) were randomized into an individual affirmation, group affirmation, or a control condition. Individual affirmation participants ranked a list of values and then wrote a short paragraph about their first-ranked value. Group affirmation participants did a similar task, focusing on the values of their ethnic group, while participants in the control condition had an unrelated task of ranking their color preferences. Participants then read a persuasive message presenting health risks related to meat consumption and the health benefits of reducing meat. Finally, they indicated their openness toward reducing meat consumption and acceptability of plant-based alternatives and lab-grown meat.

**Results and Discussion:**

Results show that affirmed participants expressed more readiness to reconsider their meat consumption habits, reduced perceptions of vegetarianism as a threat to the local culture, and more positive perceptions of the idea of lab-grown meat. However, self-esteem and frequency of meat consumption pose important limitations to the experimental effects. We discuss the findings from the perspective of self-and collective identity threats and the potential of self-affirmations to create a more open debate about animal product consumption.

## Introduction

As the saying in our country goes, you eat your vegetables and love your meat. Unfortunately, meat consumption habits are notoriously difficult to change (Macdiarmid et al., 2016), as most people believe eating plenty of meat is healthy and even necessary (Hyers, 2006; Piazza et al., 2015). However, the fact that is, for the most part, rationalized away by meat-eaters is that meat requires the slaughter and suffering of sentient beings (Singer, 1975/2009). Also, meat production threatens the environment as it contributes a great deal to air pollution (Berners-Lee et al., 2012; Shafiullah et al., 2021), as well as pollution of water, soil, and deforestation (Thornton and Herrero, 2010). What is more, empirical evidence has accumulated that relates unfavorable health outcomes to a diet rich in meat (Tilman and Clark, 2014), in particular cardiovascular disease (Abete et al., 2014) and various types of cancer (Farvid et al., 2021) As plant-based diets can be related to a deficiency of micronutrients (Kristensen et al., 2015; Schüpbach et al., 2017; Haider et al., 2018), the exact quality of vegetarian/vegan diet is crucial. However, there appears to be a growing consensus around the health benefit of reducing meat consumption, as reflected, for instance, in the latest Report on Strengthening Europe in the Fight Against Cancer (2022). Given all these arguments, the welfare of humans appears to be inextricably related to the well-being of other animals and the environment, encapsulated by the concept of One Welfare (Pinillos et al., 2016).

Although less meat would be desirable for the sake of both non-human and human animals, in most parts of the world, meat consumption is the norm (Rosenfeld, 2018; Bryant, 2019). What is more, in the contemporary world, there is a global trend toward consuming an increasing amount of meat, to so-called *meatification* of diets (Weis, 2021). Most societies provide the individual with ready-made justifications for continued meat consumption (Piazza et al., 2015), not least championed by the meat industry itself (*meatsplaining*; Hannan, 2020) In addition to being an ingrained habit, meat consumption is also embedded in the individual’s identity, social attitudes, and broader worldviews (Dhont et al., 2016; Branković, 2021). People who reduce or eliminate their meat consumption, such as vegetarians and vegans, can face negative stereotyping and even discrimination (MacInnis and Hodson, 2017; Torti, 2017; Bryant, 2019).

Previous research demonstrates, however, that humans do not readily endorse the suffering and killing of animals for meat. Especially when the link between the animals and the meat is made salient, humans experience cognitive dissonance (Loughnan et al., 2014; Bastian and Loughnan, 2017). This paper further explores the psychological mechanisms that sustain meat consumption. As a call to reduce one’s consumption of meat can threaten one’s sense of the moral self, as well as psychological needs and identities, we wanted to explore whether affirming the self could help create more psychological space for reconsidering one’s meat-eating habits. In the following, we will briefly review previous approaches to reducing meat consumption, present the self-affirmation framework and elaborate on why and how it could be applied to the matter at hand.

### Meat-reduction interventions

Previous studies demonstrate that it is possible to experimentally affect meat consumption, at least in the short term (Cordts et al., 2014; Kunst and Hohle, 2016; Carfora et al., 2017, 2019; Amiot et al., 2018; Dowsett et al., 2018; for meta-analyzes see Grundy et al., 2021; Kwasny et al., 2022). It appears from a review of the interventions that short-term outcomes, such as the immediate choice of foods, could be more amenable to change than more general habits and attitudes (Dowsett et al., 2018). Several approaches have been tested, the most frequent being providing participants with persuasive messages that informed them about health-related, ethical, environmental, or other consequences of meat consumption. Deleterious health-related effects of meat consumption appear to be the most effective persuasive arguments (Cordts et al., 2014; Carfora et al., 2019), as well as ethical arguments related to animal welfare (Auger and Amiot, 2019; Cordts et al., 2014; Kunst and Hohle, 2016). Evidence related to the noxious effects of meat production on the environment also constitutes a potentially helpful approach (Carfora et al., 2019), however, these effects are not universally present (e.g., Cordts et al., 2014). Also, combining different types of appeals could be less effective than focusing on a single category of effects (Carfora et al., 2019).

In addition to the message contents, cognitive vs. affective appeal also impacts the effectiveness of interventions (Kwasny et al., 2022). Affective aspects, e.g., negative affect related to the consumption of meat (Dowsett et al., 2018), appear to have a more prominent role than cognitive factors in shaping responses to experimental inductions. Also, matching messages with the needs of consumers (e.g., their values or decision stages) increases their effectiveness (Kwasny et al., 2022). Finally, contextual factors, such as nudging or enhancing the visibility/availability of vegetarian options, have proven helpful in encouraging meat-free options (Grundy et al., 2021; Kwasny et al., 2022).

However, there are also limits to the possibility of experimentally induced reconsideration of meat-consumption habits or their change. Some of the previous studies suggest that more complex, multi-component interventions can be effective, for instance, combining information, social norms, fear appeals, mind attribution, and self-monitoring (Amiot et al., 2018). However, some studies suggest that combining different types of appeals reduces their effectiveness (Carfora et al., 2019). Also, to induce changes in deeply ingrained habits, interventions might need to last longer and involve daily messaging to participants (Carfora et al., 2017, 2019).

Furthermore, meat identification or meat attachment (Graça et al., 2015) emerges as one the most important barriers. According to several studies, individuals that are most attached to meat, who value it for different reasons, are also those that are least receptive to counterarguments and generally least open to reconsidering their meat consumption (Allen and Ng, 2003; Dowsett et al., 2018; Roozen and Raedts, 2022). Also, as meat consumption can be closely associated with valued social identities, such as ethnic identity (Branković, 2021) or gender identity (i.e., masculinity, Rothgerber, 2013), the appeals to reduce meat have implications for how one perceives oneself. We will therefore present an intervention conceptualized within the framework of self-affirmation theory.

### Affirming the self enhances openness to persuasion

In the present study, we propose and test the potential of the self-affirmation approach to enhance openness to reconsidering meat consumption. When provided with the opportunity to affirm the general value of the self (or their valued ingroup), individuals can become more open to persuasive communications, even the ones related to health and deeply ingrained habits (Epton et al., 2015; Ferrer and Cohen, 2019).

Self-affirmation theory posits that an important motivation is to maintain a sense of self-integrity, a favorable general image of oneself, including the sense of being a moral person (Steele, 1988; Sherman and Cohen, 2006; Sherman, 2013; Cohen and Sherman, 2014). Threats to self-integrity incite defensive reactions to restore this positive image; these defensive strategies shift the attention toward the source of the threat and consume cognitive resources (Klein and Harris, 2009). Therefore, these defensive strategies diminish openness to information and the capacity for systematic information processing (Cohen and Sherman, 2014). On the other hand, an affirmation of the global sense of self prevents these defensive reactions–shifting the focus away from the specific threat, it restores the global sense of self-worth while at the same time uncoupling the threat from the self. Self-affirmed individuals are thus encouraged to a higher level of construal and perceive the threat at hand as not endangering their global sense of self-worth (Sherman, 2013). They also become more aware of their resources to deal with the threat. This process helps alleviate the consumption of cognitive resources–instead of focusing on short-term and threat-centered defenses, the individual is thus open to more constructive ways of coping with the threat, also encouraging the cycle of positive adaptive potential (Cohen and Sherman, 2014). Positive effects of self-affirmation are an approach orientation, openness to threatening information, and the possibility of using systematic processing of information (Cohen and Sherman, 2014).

Supporting the logic we outlined, experimental studies have demonstrated beneficial effects of self-affirmation procedures on openness to change of attitudes and habits related to both health behaviors (Epton et al., 2015; Ferrer and Cohen, 2019) as well as prejudice (Fein and Spencer, 1997). Furthermore, meta-analytical studies suggest that self-affirmation procedures have small but reliable effects on the acceptance of health messages, as well as heightened motivation for change and subsequent healthier behavior, across a range of health-related outcomes (Epton et al., 2015). For instance, young women at higher risk of breast cancer were more open to messages linking alcohol consumption to breast cancer after being affirmer in an unrelated domain (Harris and Napper, 2005). In addition, affirmed participants who read about the benefits of eating at least five portions of fruit and vegetables per day did report eating more fruit and vegetables at both 7-day and 3-month follow-ups compared to non-affirmed participants (Harris et al., 2014).

Self-affirmations have also been tested in the context of intergroup relations and prejudice reduction. Based on the idea that prejudice can serve self-image maintenance, a classical study revealed that self-affirmation could attenuate the tendency to stereotype out-group members after experiencing self-threat (Fein and Spencer, 1997). According to a recent review, self-affirmations prove helpful in alleviating perceived threats to valued social identities and attenuating negative intergroup relations and prejudice (Badea and Sherman, 2019). For instance, self-affirmation of values rendered participants more open to the acknowledgment of ingroup responsibility for prior conflicts, as well as support for reparative measures in post-conflict settings (Čehajić-Clancy et al., 2011).

### The role of self-affirmation for openness to meat reduction communications

How does this logic apply to meat consumption habits? We posit that persuasive communications and, more generally, interventions aimed at a reduction of meat consumption can constitute a self-threat. First, meat consumption entails killing sentient beings, a fact usually removed from the consciousness of meat eaters through dissonance-reducing mechanisms (Loughnan et al., 2014; Bastian and Loughnan, 2017). However, when this fact is made salient, a threat to the sense of one’s morality can entail. Previous research usually documents this threat through defensive mechanisms that are being put into action. Culturally supported and transmitted legitimizations for meat consumption (Hyers, 2006; Piazza et al., 2015) also attest to the need to deter the threat to one’s morality. However, other dissonance-reducing strategies can also be engaged, such as a change in attitudes toward meat consumption and the consumption behaviors themselves (Kunst and Hohle, 2016).

In the present research, we will build upon the distinction made in the previous research between individual and group affirmations (Sherman et al., 2007). In addition to affirming the individual sense of integrity and morality, an affirmation can also target a collective self-image or a social identity. Although, as evidenced by previous research, the individual affirmations appear to be more effective (Badea and Sherman, 2019), we tested both types of inductions since at least one of the barriers to meat reduction communications is related to social identity.

Previous studies found that meat eaters can experience moral reproach, that is, the expectation that vegetarians and vegans will judge them for their meat consumption (Minson and Monin, 2012; Branković and Budžak, 2021). This concept of moral reproach thus encapsulates the perceived threat to the moral self, and we hypothesize that providing the opportunity for self-affirmation works through alleviating the expectation of moral reproach.

On the other hand, as meat consumption can be closely associated with gender or ethnic identity, reconsidering one’s meat consumption habits can entail a social identity threat. As demonstrated by previous research (Branković, 2021), meat consumption and general attitudes toward animals are predicted by one’s attachment to the ethnic group. This relationship is mediated by the perceived threat of vegetarianism to traditional cultural values and ways of life. In line with this, experimentally reinforcing the link between abstinence from meat and the religious tradition of fasting helped improve attitudes toward vegetarians in general (Budžak and Branković, 2022). We thus expected that another mediator of self-affirmation (more precisely, group affirmation) would be a decreased sense of cultural threat related to reducing meat consumption.

Thus, given the argumentation that a call to reduce one’s meat consumption can constitute a relevant self-threat, we propose that affirming the global sense of self-worth can help alleviate these threats and increase openness to meat reduction advocacy. It has been pointed out that self-affirmation procedures do not remove the underlying cause of prejudice (Badea and Sherman, 2019), and we concur that they cannot in themselves sway either attitudes or habits related to meat consumption. However, if they can effectively alleviate the threat to the individual or social identity, this could help „unfreeze“potential barriers and create more space to engage in systematic processing and consider valid arguments (Cohen and Sherman, 2014). The reviewed literature supports the potential of self-affirmation procedures to incite more openness to both health–and nutrition habits (Harris et al., 2014; Epton et al., 2015; Ferrer and Cohen, 2019) as well as to reduce perceived threats to valued social identities (Branković, 2021).

## The present study

**Figure 1:**
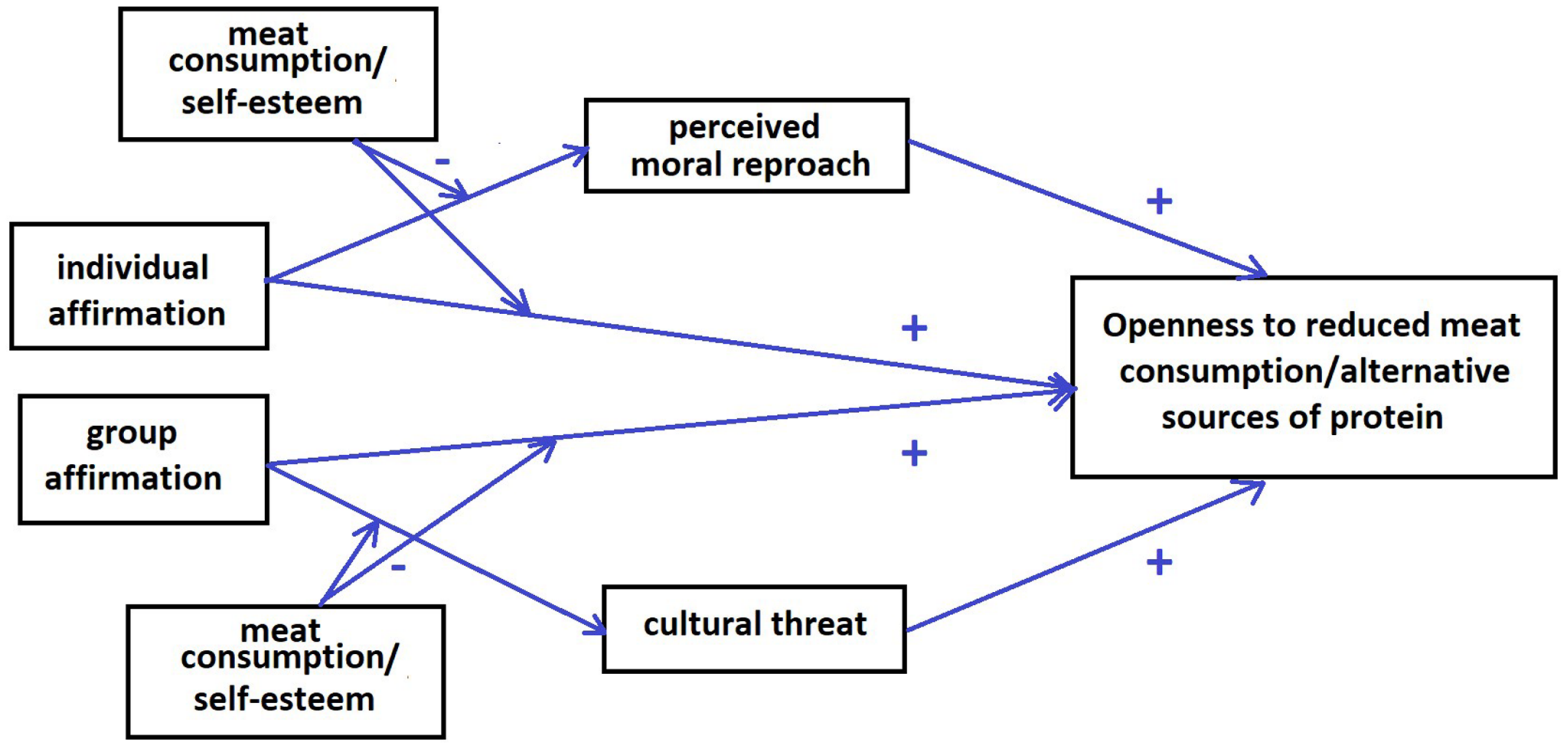
The theoretical model: The moderators and mediators of the effect of self-affirmation on the openness to meat reduction/alternative protein sources.

In Figure 1, we summarize the theoretical model in which we propose that:

1. individual and group affirmations can increase openness to reduce meat consumption and consider alternative, plant-based sources of protein and lab-grown meat (Cohen and Sherman, 2014; Epton et al., 2015; Ferrer and Cohen, 2019),
2. individual affirmation would work through a reduced sense of moral reproach (Branković and Budžak, 2021), while group affirmations would reduce the perceived cultural threat (Branković, 2021), thus leading to more openness toward meat reduction communications,
3. frequency of meat consumption would negatively impact the capacity of affirmations to create more openness, that is, we expect more frequent meat eaters to be less susceptible to these inductions (Graça et al., 2015).

## Materials and methods

### Design

Participants were randomized into three conditions: individual affirmation, group affirmation, and no affirmation (control group). The affirmation inductions are specifically value-affirmations (McQueen and Klein, 2006). Participants were asked to rank a list of 10 values and then briefly describe a situation or example that shows how the first-ranked value is reflected in their personal (or group) experience. Participants in the control group were presented with identical tasks related to their color preferences. The experiment was pre-registered at https://aspredicted.org/km3jh.pdf.

### Participants

We aimed to recruit at least 246 participants in total, as suggested by power analysis, to be able to record moderate-size effects with the power of 0.8, with the alpha level set at 0.05, using G*Power (Faul et al., 2009).

We applied two exclusion criteria: a. we excluded participants who failed to answer to the questions about values ranking and value description, as they constituted the experimental induction, b. we excluded participants who failed to answer at least 50% of the dependent variable items. We did not exclude the participants who ranked all of the values and wrote at least something in answer to the follow-up questions, however unelaborated (there were 14 such participants), as the unelaborated answers do not indicate a failure to consider the issue but rather reluctance to write complex answers. In addition, we excluded from further analyzes six participants who self-declared as vegans or vegetarians (i.e., who reported not eating any fish or meat) as the dependent variables are not meaningful measures for them. The analyzes were replicated on the whole sample, including the vegetarians and vegans, and the results were not changed.

We recruited participants using the passive snowball method (Parker et al., 2019), though sharing the link for the experiment on social media. The final sample thus included 271 participants, 66% women, aged from 17 to 65 (*M* = 30.24, SD = 11.42). The sample included individuals who graduated from primary school (3%), high school (16.6%), students (45%), and individuals who graduated from faculties (29.9%) or post-graduate studies (8.5%). Participants were fairly equally distributed among the conditions; there were 88 participants in the individual affirmation, 94 in the group affirmation, and 89 in the control group. Participants were randomly assigned to the conditions.

Participants were provided with informed consent prior to participation. They were informed about the general research question, that their participation was voluntary and anonymous and that they could withdraw from further participation at any time. Participants were debriefed directly after participation and were provided with the contact of the principal investigator in case they had any questions.

The study was approved by the Institutional Review Board of the Department of Psychology of the Faculty of Media and Communications in Belgrade.

### Procedure

#### The affirmation inductions

Participants in the individual/group affirmation conditions were given a list of 10 values and instructed to rank them according to their personal priority/importance to their ethnic group. We presented the following values: living in the present moment, being connected with friends and family, trust among people, loyalty and integrity, religious values, solidarity in society, sense of humor, contribution to society, democracy, and creativity. All the research team members assessed whether the values related to the threat domain and such values were excluded from the list.

After having ranked the values, we followed up with the instruction to briefly explain why the first-ranked value is of personal/group importance, for instance, to describe a situation from the personal experience or a historical event that clearly demonstrated how it is important for the individual or the group. The Control group had an identical task, except that participants ranked favorite colors and explained how this color is personally appealing, e.g., to describe an object or context in which it was appealing.

#### The vignette

Thereafter, we presented participants with a vignette briefly introducing health-related risks of meat consumption (e.g., higher incidence of disease), benefits of decreased consumption, as well as alternative sources of protein, including lab-grown meats. After that, participants indicated their interest in learning more about the topic, their readiness to consider their meat-eating habits, reduce their consumption of meat in the following 6 months, and how they perceive the idea of consuming alternative plant-based proteins and lab-grown meats. Participants in all three conditions read the same vignette.

Participants were instructed to carefully read the vignette as we announced that we would ask them some questions about the text at a later point. The vignette was presented in a form resembling a journal article and was 440 words long. We opted to focus on the health-related risks of meat consumption and the benefits of decreased consumption, as we believed the information could be perceived as relatively personally significant for meat-eaters. This belief is reinforced by previous studies that found that health-related messages were successful in affecting attitudes toward meat consumption (Carfora et al., 2019). To make a case for meat reduction sufficiently persuasive, we referenced a paper presenting results of medical research documenting the risks of meat consumption, as well as a nutrition expert and microbiologist to explain the logic behind the idea of lab-grown meat. The translation of the vignette is available at https://osf.io/am9xe/.

Also, we introduced the idea that meat-based protein can be replaced with plant-based sources of protein (e.g., mushrooms, peas, beans, and greens). Finally, we briefly introduced the idea of lab-grown meat, that is, how it is being manufactured and that this would be a healthier and more ethical alternative for those who wish to continue eating meat.

### Measures

#### Ratings of the vignette

After reading the vignette, participants indicated on 10-point rating scales, ranging from 1 (*not at all*) to 10 (*very much*) the extent to which they thought the text was interesting, understandable, informative, persuasive, meaningful, thought-provoking, and stylistically accomplished. We computed a single mean score as the ratings made an internally consistent scale (*α* = 0.91).

#### Openness to meat reduction

After that, the participants indicated on 10-point scales their (a) interest in learning more about the topic, (b) their readiness to reconsider meat consumption habits, (c) their readiness to reduce meat consumption in the following 6 months, (d) the acceptability of the idea of plant-based protein, and e. the acceptability of the idea of lab-grown meat. We conducted a principal component analysis on the items, which yielded one component that explained 70% of the variance and had high loadings from all of the items (the minimum was 0.664). We computed a mean score of openness to meat reduction (*α* = 0.89).

*Perceived moral reproach against non-vegetarians* was measured by two items rated on 7-point scales (*α* = 0.82), specifically: “Vegetarians mostly consider non-vegetarians immoral” and “If they saw me eat meat, most vegetarians would consider me immoral” (Branković and Budžak, 2021).

*The cultural threat scale* was based on the Vegetarianism threat scale developed by Dhont and Hodson (2014), which was translated into Serbian and previously used in research in the local context (Branković, 2021). We chose the three items with the highest factor loadings for the current study, namely: “Vegetarianism poses a threat to our country’s customs and traditions,” “Vegetarians and vegans should have more respect for the local traditional cuisine, which is simply based on meat,” and “People who insist on a vegetarian/vegan diet spoil important family gatherings and celebrations” (*α* = 0.73). We also changed the target group of the items to include both vegetarians and vegans, as previous research shows that vegans could be perceived as a more threatening group than vegetarians (Branković and Budžak, 2021).

### Controls

Participants chose what best described their eating habits from the following options: (a) “I consume meat regularly,” (b) “I consume meat, but try to decrease the intake,” (c) “I consume meat only occasionally,” (d) “I consume fish, but not other types of meat,” (e) “I do not consume meat, but consume other animal products (dairy, eggs),” and (f) “I never consume meat or any products of animal origin.” The item was reverse-coded so that a higher score indicates more frequent meat consumption. The measure was previously validated for the local context (Branković, 2021). The labels (e.g., omnivore, vegetarian, vegan) were intentionally omitted, as they can be understood differently by respondents (e.g., some people who claim to be vegetarian eat meat, and some people who do not eat meat prefer not to be called vegetarians). Most of our participants consume meat regularly (54.6%), 23.2% reported that they consume meat but try to decrease the intake, 18.8% consume meat occasionally, 3.3% consume fish but no other types of meat, 1.1% do not consume meat, but consume other animal products, and 1.1% never consume meat or any other products of animal origin.

Self-esteem was measured by a translated and adapted version of the scale devised by Tafarodi and Swann (2001), capturing the two aspects of a global self-evaluation, self-competence, and self-liking, e.g., “When I think about myself, I feel great.” or “I never doubt my own worth”. Sixteen items were rated on a 5-point scale, anchored from 1 (*do not agree at all*) to 5 (*completely agree*). We computed a single global score as the items had high inter-correlations (*α* = 0.90).

To establish the strength of ethnic identification, participants indicated how personally important they felt belonging to their ethnic group was on a scale from 1(*not at all important*) to 5 (*highly important*). The validity of the single-item measure was established in previous research in the region (Branković et al., 2020).

### Analytical strategy

As pre-registered, ANOVA was used to test the differences between groups, whereas planned contrasts constituted the main tests of the hypothesis: we compared the individual and the group-affirmation condition against the control condition (1 1–2); after that, the individual and the group-affirmation condition were contrasted (1–1 0), to test for possible differences in their efficiency.

## Results

### Descriptive statistics

Descriptive statistics and the correlations of the variables are presented in Table 1. The mean level of self-esteem is somewhat above the theoretical mid-point, as expected from prior research. However, the level of ethnic identification, perceived vegetarianism threat, and the perceived moral reproach against meat-eaters are somewhat below the midpoint of the scale, indicating that these perceptions are not overly strong in the current sample.

**Table 1:** Descriptive statistics and correlations of the measured variables (*N* = 271).

	*M*	SD	2	3	4	5	6	7
1. Self-esteem (1–5)	3.50	0.65	0.023	0.055	−0.034	−0.076	−0.160**	0.084
2. Ethnic identification (1–5)	2.60	1.33		0.215**	0.012	0.045	−0.053	0.048
3. Cultural threat (1–7)	2.38	1.40			−0.15*	−0.112	−0.153*	0.096
4. Perceived moral reproach (0–100)	35.53	22.89				0.061	0.114	−0.170**
5. Ratings of the vignette (1–10)	7.47	1.86					0.706**	−0.229**
6. Openness to meat reduction (1–10)	5.87	2.52						−0.383**
7. Frequency of meat consumption (1–4)	3.29	0.87						

*We report mean (*M*) and standard deviations (SD) for all of the variables measured in the first two columns, in the following columns we report their inter-correlations. *N* refers to the sample size. *p < .05, **p < .01.

The vignette was rated quite favorably, and the follow-up analysis suggests that the ratings did not differ across the experimental conditions, *F* (2, 268) = 0.308, *p* = 0.736. The overall index of openness to meat reduction indicated moderate levels of such openness. In Table 2, we present in more detail the expressed interest in and preparedness to reconsider meat reduction and the acceptability of alternative protein sources. We can see that participants are moderately open to both these ideas, most to considering the alternative, plant-based sources of protein and least to the idea of lab-grown meat. Notably, more frequent meat-eaters rated the vignette less favorably and expressed less openness to meat reduction in general.

**Table 2:** Openness to meat reduction and alternative protein sources (*N* = 271).

	*M*	SD
Interest in learning more about the topic	6.08	2.83
Readiness to re-consider meat consumption habits	6.21	3.03
Readiness to reduce meat consumption in the following six months	5.92	3.15
Acceptability of the idea of plant-based protein	6.70	2.95
Acceptability of the idea of lab-grown meat	4.48	3.16

### Control variables

Participants in the three conditions did not differ significantly in terms of their level of self-esteem, *F* (2, 268) = 0.68, *p* = 0.519, the strength of ethnic identification *F* (2, 268) = 0.51, *p* = 0.600, or the frequency of meat consumption, *F* (2, 268) = 2.43, *p* = 0.090.

### Test of the pre-registered hypothesis

Omnibus ANOVA did not yield significant differences, *F* (2, 265) = 1.89, *p* = 0.153; however, the planned contrasts described previously helped clarify these results. First, the individual and group affirmation conditions combined were significantly different compared to the control group, *t* (265) = 1.90, *p* = 0.029. In contrast, the individual and the group affirmation conditions were not significantly different from each other, *t* (265) = 0.443, *p* = 0.329. Thus, the affirmed participants expressed somewhat more openness to meat reduction and alternative sources of protein (the individually affirmed *M* = 6.16 (*SD* = 2.38), group affirmed *M* = 6 (SD = 2.72), control group *M* = 5.46 (SD = 2.39)).

### Test of the moderated mediation model

As presented in Figure 1, we hypothesized that the individual affirmations would decrease perceptions of moral reproach, while the group affirmation would decrease perceptions of cultural threat. As we revealed correlations between the outcome variable with self-esteem and the frequency of meat consumption, we also wanted to test their potential role as the moderators of the experimental inductions: people with higher self-esteem and more frequent meat consumers would be expected to be less susceptible to the affirmations.

To examine these relationships more closely, we conducted moderated mediation analysis using SPSS Process software (Hayes, 2013).

First, self-esteem did not moderate the effects of the individual affirmation on openness to meat reduction, [*b* = −0.19, SE = 1.61, 95%CI (−5.10, 1.26)] nor did the frequency of meat consumption moderate the effects of the group affirmation on openness to meat reduction, [*b* = −0.0.02, SE = 0.01, 95%CI (−0.15, 0.12)]. We also conducted a post-hoc power analysis using G*Power software (Faul et al., 2009), to check whether we had sufficient power, given the small effects. The analysis suggested 65% probability to detect a moderating effect of self-esteem and 42% probability to detect a moderating effect of the frequency of meat consumption. Having in mind the insufficient observed power, we proceeded to test the simple mediation models. Mediation was tested with 5,000 bootstrap samples, and two separate analyzes were conducted for the two presumed paths (individual affirmation to openness via moral reproach; group affirmation to openness via cultural threat perceptions).

The individual affirmation induction did not affect the perceived moral reproach, [*b* = 1.25, SE = 1.00, 95%CI (−0.71, 3.22)]. However, the perceived moral reproach had a significant effect on openness to meat reduction [*b* = 0.01, SE = 0.1, 95%CI (0.00, 0.03)]. When moral reproach was entered into the model, the induction ceased to be a significant predictor, [*b* = 0.13, SE = 0.11, 95%CI (−0.09, 0.34)]. However, we cannot conclude that the mediation is significant since the first path is not.

Similarly, group induction did not affect the perceptions of cultural threat, [*b* = −0.08, *SE* = 0.06, 95%*CI* (−0.20, 0.04)], whereas the perceptions of cultural threat had a marginally significant effect, (*b* = −0.26, *SE* = 0.11, 95%*CI* [−0.48, −0.05]). When moral reproach was entered into the model, the induction ceased to be a significant predictor, [*b* = 0.04, *SE* = 0.11, 95%*CI* (−0.17, 0.25)]. However, we cannot conclude that the mediation is significant since the first path is not.

Thus, our analyzes did not support the role of either perceived moral reproach or perceptions of cultural threat as mediators of the experimental inductions. Further, individual differences in self-esteem and the pre-induction frequency of meat consumption proved to have a generally negative effect on the openness to meat reduction. Their effects suppressed the effects of the experimental inductions, thus indicating significant barriers to meat reduction advocacy.

## Discussion

In the current study, we presented our participants with an opportunity to affirm their individual or group values, and after that, they were given valid arguments to reconsider meat consumption. We expected that the self-affirmation procedure would help alleviate the self-relevant threats (e.g., to the morality of self or valued group traditions and identities), thus rendering participants more open to arguments about ethical issues and health-related risks of meat consumption. Our inductions produced small but significant effects, such that both affirmed groups expressed more openness to meat reduction than the control group. However, we also established that people with higher self-esteem and more frequent meat eaters are generally less open to reconsider their meat consumption, regardless of whether they are presented with persuasive messages.

Our findings align with previous research that found small positive effects of similar affirmation procedures on health–and nutrition-related habits (Epton et al., 2015; Ferrer and Cohen, 2019). However, as some previous reviews suggested (Badea and Sherman, 2019), we did not find a significant difference in the effectiveness of the individual over group-based affirmations. In our study, the expressed openness to meat-reduction arguments was highly correlated with the ratings of message persuasiveness (0.70). However, ratings of the message did not significantly differ between the experimental groups and the control group. Thus, we do not have sufficient evidence to conclude that the affirmation procedures increased the capacity for systematic processing, as suggested by the theoretical framework (Cohen and Sherman, 2014).

The presented findings thus support further study of affirmation procedures for meat-reduction communications. However, our findings also suggest at least three important caveats.

First, the effects we captured are small, and several characteristics of the persuasive message we devised could be relevant to this finding. The arguments presented were rated quite highly, so this might have produced a ceiling effect in attitude change. We opted for offering strong arguments, as the general idea behind self-affirmations is to incite more positive reactions only when valid arguments are presented. This is also ethically more acceptable as presenting weak arguments could lead to even more entrenched attitudes about the benefits of meat consumption. However, given the presumed ceiling effect, perhaps larger effects of the induction would be detected in case argument quality were also manipulated (*cf.* Petty and Cacioppo, 1986). For instance, a control group of participants who are not offered any arguments could be included to establish the base-rate reaction of meat-eaters. As even the control group read the vignette that presented valid and persuasive arguments in favor of meat reduction in the present study, this constituted a fairly strict test of the effectiveness of self-affirmation. Another relevant aspect of the message is that the arguments were supported by citing scientific sources. Perhaps larger resistance and larger potential benefits of affirmation could be expected if it were ascribed to an out-group source, e.g., a vegan activist (Hornsey and Imani, 2004).

Further, regarding the effectiveness of the individual and group-based affirmation, the current framework did not allow for a more precise matching of the underlying motivations for meat consumption, identities, and perceived threats related to meat reduction. As all of these characteristics can vary between individuals (Rosenfeld and Burrow, 2017), individual affirmations could be more relevant for those sensitive to individual self-image threats, e.g., the threat to morality. In contrast, group affirmations would be more effective for individuals with a stronger sense of cultural threat or a stronger ethnic identification. Future studies could attempt such participant-message matching to be able to identify the most promising affirmation procedures. Moreover, specific rhetoric strategies used by the animal agriculture industry should be studied within this framework for the most ecologically valid conclusions (Hannan, 2020).

In terms of the underlying processes through which affirmation procedures work to unfreeze attitudes, we hypothesized that individual affirmation would work to alleviate the perceived moral reproach related to meat consumption (Minson and Monin, 2012) while group affirmation would work through a decreased perception of cultural threat (Branković, 2021). However, while both mediators did have a significant relation to the openness to meat reduction, neither was affected by the experimental inductions. One possible reason for this is the nature of the persuasive message we used. Specifically, the arguments presented were mainly related to health risks and benefits. Since we only touched upon the ethical issues and did not consider the social and cultural context of meat consumption in the message, perhaps the underlying reasoning or affective processes were not sufficiently generalized to these issues, as their relationship had not been made salient. Thus, future studies need to conceptualize the procedures and mediating processes at a more specific level.

Finally, our findings show that there are important limitations as to the effectiveness of affirmation procedures, and both self-esteem and the previous frequency of meat consumption emerged as significant negative predictors of the openness to meat reduction. Previous literature suggests that mostly lower self-esteem recipients can experience the strongest effects of self-affirmation procedures (McQueen and Klein, 2006). Our findings are generally consistent with this, although we did not have sufficient power to detect a moderation effect. We presume this is because participants with higher self-esteem typically have more effective defensive strategies and, therefore, can rely on their own psychological resources to alleviate self-threats that emerge in meat-reduction communications. Alternatively put, lower self-esteem participants could experience the most benefit from self-affirmation. This point is to be further corroborated in future research.

Further, more frequent meat eaters are also less susceptible to persuasion attempts, even when the arguments are valid and they have been provided with the opportunity to affirm their self-integrity. Such resistance is interpretable as an effect of personal involvement and the consequent biased processing (Kunda, 1990; Zuwerink and Devine, 1996) and is in line with previous studies demonstrating the role of meat attachment (Graça et al., 2015; Roozen and Raedts, 2022). Presumably, frequent meat eaters are more motivated to be defensive toward meat-reduction communications, but they could also have more elaborated or stronger counter-attitudes or justifications (Piazza et al., 2015). In effect, self-affirmation does not appear as a promising path to unfreezing attitudes in this group of participants. For them, other approaches should be tested, such as the availability of plant-based options that could be shown as sufficiently attractive in the first place (Lehner et al., 2016; Kurz, 2018) or paradoxical interventions that have proven useful in unfreezing resistant attitudes in different domains (Bar-Tal and Hameiri, 2020). Furthermore, it has been proposed that meat justifications can be rooted in a broader irrational worldview (Hannan, 2020), and potential underlying irrational beliefs should be further studied.

Despite the limitations, our study does lend preliminary support to the usefulness of self-affirmation procedures, although their effects should be specified through further research. Presumably, self-affirmations are to be combined with other interventions to be effective, for instance, as a first step in creating room for discussion. In the present study, we opted for the most commonly used procedure to induce affirmation, writing about one’s values (McQueen and Klein, 2006). However, other procedures might be more suited for the issue of meat reduction and should be further tested. We also relied on health-related arguments for meat reduction, but other types of content and arguments should also be investigated. Given the predominance of the health-related association in the perception of vegetarians and vegans suggested by previous research (Branković and Budžak, 2021), we thought this would constitute relevant content for the persuasive message. However, ethical and moral issues could be the ones most directly related to self-integrity and the experienced self-that (Cohen and Sherman, 2014), so perhaps self-affirmations could play an even more prominent role in such cases.

Against the general backdrop of meat-reduction efforts and barriers, this study is the first to test the potential of the self-affirmation approach. Given the importance of meat reduction to the well-being of humans, other animals, and the environment (Pinillos et al., 2016; Weis, 2021), we hope these findings help understand and devise more effective policies and communications and inspire further research.

## Data availability statement

The original contributions presented in the study are included in the article/supplementary material, further inquiries can be directed to the corresponding author.

## Ethics statement

The studies involving human participants were reviewed and approved by Institutional Review Board of the Department of Psychology of the Faculty of Media and Communications, Singidunum University in Belgrade. The patients/participants provided their written informed consent to participate in this study.

## Author contributions

MB, AB, NT, and JJ contributed to the study’s conceptualization, design, and data collection. MB conducted the analyzes and prepared the manuscript. All authors contributed to the article and approved the submitted version.

## Funding

This research was supported by the European Association of Social Psychology extraordinary grant, Social Psychology Ambassadors, awarded to the MB.

## Conflict of interest

The authors declare that the research was conducted in the absence of any commercial or financial relationships that could be construed as a potential conflict of interest.

## Publisher’s note

All claims expressed in this article are solely those of the authors and do not necessarily represent those of their affiliated organizations, or those of the publisher, the editors and the reviewers. Any product that may be evaluated in this article, or claim that may be made by its manufacturer, is not guaranteed or endorsed by the publisher.
